# Multisensory Motion Perception in 3–4 Month-Old Infants

**DOI:** 10.3389/fpsyg.2017.01994

**Published:** 2017-11-15

**Authors:** Elena Nava, Massimo Grassi, Viola Brenna, Emanuela Croci, Chiara Turati

**Affiliations:** ^1^Department of Psychology, Università degli Studi di Milano Bicocca, Milan, Italy; ^2^Department of Psychology, University of Padua, Padua, Italy

**Keywords:** motion, amodal, visuo-tactile, audio-visual, development, multisensory

## Abstract

Human infants begin very early in life to take advantage of multisensory information by extracting the invariant amodal information that is conveyed redundantly by multiple senses. Here we addressed the question as to whether infants can bind multisensory moving stimuli, and whether this occurs even if the motion produced by the stimuli is only illusory. Three- to 4-month-old infants were presented with two bimodal pairings: visuo-tactile and audio-visual. Visuo-tactile pairings consisted of apparently vertically moving bars (the Barber Pole illusion) moving in either the same or opposite direction with a concurrent tactile stimulus consisting of strokes given on the infant’s back. Audio-visual pairings consisted of the Barber Pole illusion in its visual and auditory version, the latter giving the impression of a continuous rising or ascending pitch. We found that infants were able to discriminate congruently (same direction) vs. incongruently moving (opposite direction) pairs irrespective of modality (Experiment 1). Importantly, we also found that congruently moving visuo-tactile and audio-visual stimuli were preferred over incongruently moving bimodal stimuli (Experiment 2). Our findings suggest that very young infants are able to extract motion as amodal component and use it to match stimuli that only apparently move in the same direction.

## Introduction

The past two decades have seen a growing interest in the development of multisensory processing (e.g., see Bremner et al., 2012), particularly driven by studies suggesting that human infants, just like adults, begin very early in life to take advantage of multisensory information to better detect and discriminate events (Bahrick et al., 2004; Lewkowicz and Kraebel, 2004). In particular, it appears that infants are able to extract the invariant amodal information that is conveyed redundantly by multiple senses, such as space, time, intensity, rhythm and tempo (Bahrick and Lickliter, 2002), likely because the dimensions of time and space are naturally correlated (De Hevia et al., 2014). For instance, when we look at a talking face we perceive specific attributes conveyed by the single senses, such as the color of the skin or hair (i.e., visual features), and the pitch and timbre of the voice (i.e., auditory features). However, we know that these features belong/come from the same person because they occur in the same time (i.e., synchronously) and space.

Temporal synchrony is known to be the amodal information that infants use the most to bind multisensory input (Lewkowicz, 2000), which could promote, among the other, audio-visual speech perception (Gogate and Bahrick, 1998; Gogate et al., 2000; Baart et al., 2014). That is, infants’ ability to extract the amodal component from multimodal input allows them to perceive events as unitary and coherent. Because amodal information is invariant across multiple senses (e.g., a rhythm can be heard, seen or felt on the skin), it is not surprising that infants’ sensitivity to multisensory events has been found for different sensory combinations. For instance, studies have shown that, by 3 months of age, infants match audio-visual events, such as moving objects and their impact sounds (Bahrick, 1988; Lewkowicz, 1992), their distal relations (e.g., detection of dynamic auditory-visual correspondences for movements in the near-far plane, see Pickens, 1994), and the rhythm they convey (Bahrick and Lickliter, 2004). Moreover, studies on crossmodal transfer show that even newborns can detect/extract amodal properties such as the shape and the texture of objects from one modality and transfer them in a different modality, e.g., from vision to touch (see Sann and Streri, 2007) or from hand to eye (Streri and Gentaz, 2003, 2004). Visual-tactile correspondences are likely promoted by infant’s exploration of their own body, which represents the first and important source of multisensory stimulation, as infants continuously move and act on their own body by touching it and looking at it (Rochat, 1995). This has recently been corroborated by studies showing that newborns prefer to look at a baby face being synchronously stroked with their own face than asynchronously, suggesting that intersensory synchrony is fundamental for the development of body representation (Filippetti et al., 2013).

According to the Intersensory Redundancy Hypothesis (IRH, Bahrick et al., 2004), the detection of these amodal relations guides attention toward multisensory events and objects; it is thus the amodal property of the multimodal, redundant stimuli to promote integration among the senses. In other words, early in development, processing and learning of amodal properties is facilitated in multimodal stimulation. In contrast, processing and learning of modality-specific properties is facilitated when information is experienced unimodally. As perceivers become more experienced, perceptual processing becomes increasingly flexible, such that amodal and modality-specific properties are detected in both unimodal and multimodal contexts (Bahrick and Lickliter, 2004; but see Tomalski et al., 2013, underlying the possibility that this model may apply until 8 months of age particularly for language acquisition).

Another feature that appears to promote multisensory processing is motion. For example, to observe whether infants can extract tempo and rhythm from multisensory stimulation, several studies have used a visual moving object (e.g., a hammer, see Bahrick et al., 2002, 2004) accompanied by a sound. Movement facilitates the processing of objects and events and is attractive for infants. Earlier studies have indeed shown that young infants not only prefer moving to static objects (Otsuka and Yamaguchi, 2003), but that movement promotes the development of different perceptual skills, including recognition of unfamiliar faces (Otsuka et al., 2009), object unity (Kellman et al., 1986; Johnson and Aslin, 1996), and sensitivity to figural coherence in displays of biomechanical motions (Bertenthal et al., 1984).

Despite extensive literature about infants’ processing of both multisensory and dynamic information, evidence regarding infants’ sensitivity to the coherent direction of audio-visual moving events is still limited and controversial. Bremner et al. (2011) found that 2-, 5-, and 8-months of age infants were able to detect the dynamic co-location of an audio-visual event. A ball moved along the horizontal plane and a sound was presented stereophonically via two hidden speakers creating the impression of a sound moving at the same speed of the ball (this was achieved through varying the balance from equal volume at each speaker). Indeed, after habituation to the co-located audio-visual display, all age groups showed sensitivity to the violation of auditory-visual matching of dynamic spatial co-location. However, it is interesting to note that, when presented with the stimuli without previous habituation, infants of all ages did not show any spontaneous preference for either the co-located or non-colocated stimuli, suggesting that audio-visual co-location requires learning. However, the absence of preference may have depended upon the position of the stimuli, which were moving along the horizontal plane. It has been documented that free-field sound localisation substantially develops between 5 and 12 months of age (Ashmead et al., 1991), thus it could be that even 8 month-old infants are still developing the ability to discriminate a sound moving on the horizontal plane. This possibility may sound reasonable, considering some recent findings, in which 3 month-old infants, presented with vertically moving stimuli, were found to prefer a dot moving upward or downward on a screen, accompanied by a sound moving coherently in pitch (a tone moving up and down in frequency, see Walker et al., 2010; Dolscheid et al., 2014). This suggests that manipulating the horizontal vs. vertical space, onto which stimuli are presented could promote the preference for congruent multisensory motion perception. It should nonetheless be noted that these studies (Walker et al., 2010; Dolscheid et al., 2014) included, as pointed out by Lewkowicz and Minar (2014), not only a change in the sound pitch, but also a change in the sound intensity. That is, the infants’ preference for congruent audio-visual motion signals reported might have reflected a sensitivity to pitch-loudness interactions, rather than to a genuine sensitivity to the modulation of the sole pitch. In turn, this would imply that infants did not react to the congruency/incongruency of the auditory and visual motion, extracting motion as an amodal property of audio-visual moving stimuli, but to the modulation of the intensity of the auditory stimulus.

In order to overcome the limitations of previous literature, here we investigated whether infants can extract motion as amodal component across different sensory combinations, using visual and auditory stimuli in which motion is only apparent because induced by a perceptual illusion. Specifically, audio-visual associations were examined using the so-called Barberpole illusion, in both its visual and auditory version. In the visual version of the Barberpole illusion, when a one-dimensional diagonally moving grating is presented within a rectangular aperture, the two-dimensional line terminators at the edges of the aperture bias the perceived direction of motion, giving thus the impression of upward/downward motion. In the auditory version, a sound is perceived as endlessly moving upward (or downward) in auditory pitch. Crucially, in both the visual and auditory version of this illusion space and time are not manipulated, nor other sensory features, such as loudness. Importantly, the Barberpole illusion allows the motion to be perceived as continuous, with no change in direction (see Nava et al., 2016); that is, contrary to previous studies (Walker et al., 2010; Bremner et al., 2011; Dolscheid et al., 2014), the stimuli did not give the impression of bouncing from one side of the screen to the other, but were perceived as constantly moving in the same direction. We thus reasoned that this type of presentation would facilitate the processing of the stimuli and therefore reveal a preference for the congruency. Moreover, we investigated whether the infants’ sensitivity to multimodal dynamic events can be extended to visuo-tactile associations, which do not necessarily require manipulations of the intensity of the stimuli events, and also share the same spatial dimension (i.e., they both move along the vertical dimension). This was achieved by manually stroking the infant’s back with a paintbrush in either the same or opposite direction with respect to the visual stimulus.

Experiment 1 addressed whether infants can learn to discriminate congruent and incongruent audio-visual and visuo-tactile moving events following habituation to either condition (congruent and incongruent). Experiment 2 addressed whether infants spontaneously prefer congruent over incongruent audio-visual and visuo-tactile moving stimuli given a fixed time to process them. We hypothesized that if infants prefer congruent over incongruent moving stimuli – at net of their discrimination abilities – it would prove that complex motion perception may represent an early developing feature perceived across sensory modalities.

## Experiment 1

### Method

#### Participants

Twenty-four 3–4-month-old infants (9 females, mean age = 120 days, range = 94–134) took part in this experiment. Three additional infants were excluded from the final sample because they started crying during testing. Infants were recruited via a written invitation, which was sent to parents based on birth records provided by neighboring cities. Infants had no visual, auditory or tactile abnormalities, as reported by the parents on the questionnaire administered at the end of the testing. No infant was at risk for developmental delay or disability (e.g., pre-term, low birth weight).

Both parents signed an informed consent prior to the beginning of the experiment. The study was approved by the Ethical Committee of the University of Milan-Bicocca, and carried out in accordance with the provisions of the World Medical Association Declaration of Helsinki.

#### Materials

Visual stimuli consisted of sinusoidal gratings alternating the white and red colors moving behind an elongated rectangular aperture, leading to the impression of a movement along the major axis of the aperture (the so-called “barber-pole illusion”) and presented on a black background. Stimuli were presented on a 24-inch monitor (52 cm × 32 cm). The size of the aperture was 9.5 cm × 2.5 cm, the spatial frequency of the gratings was 0.8 cpd, and they moved at ca. 0.75°/second. The direction of motion inside the aperture was perpendicular to grating orientation and all gratings moved in the same direction at 45°.

Tactile stimuli consisted of a paintbrush that was used to stroke the back of the infant. The movements were performed from the lowest part of the back to the neck or from the neck to the lowest part of the back in only one direction at a time (i.e., the infant was stroked only upward or downward within the single trial).

Auditory stimuli consisted of several tones gliding continuously in frequency, (i.e., the so-called “Shepard-Risset glissando,” a variation of the ‘Shepard scale,’ Shepard, 1964). The stimulus consisted of a superposition of nine harmonic tones each gliding log-linearly in frequency. The amplitude of each sine wave was modulated with a bell-shape Gaussian envelope thus creating the auditory illusion of a complex tone that continually ascends (or descends) in pitch (also called the “sonic barber-pole”). The frequencies of the stimulus ranged between 275 and 8000 Hz and were presented at a constant intensity level (ca. 60 dB SPL) from two loudspeakers positioned at the sides of the monitor. All stimuli had a maximum duration of 60 seconds. Visual and auditory stimuli were programmed and presented using the Psychtoolbox of Matlab 2013a.

The modality combinations presented were visuo-tactile and audio-visual. For each condition (i.e., visuo-tactile and audio-visual) there was a congruent and an incongruent pairing. ‘Congruent’ meant that the direction of motion was similar in the two sensory modalities, while ‘incongruent’ meant that the direction of motion was opposite in the two sensory modalities.

#### Procedure

The infants sat on the experimenter’s lap who was blind to the experiment in a research room within the University of Milan-Bicocca. The caregiver could stay next to the infant for the whole duration of the experimental session and could decide to stop the testing in case s/he felt the infant was uncomfortable. The distance between the monitor and the infant was approximately 1 m and the infant’s eye level was aligned to the center of the screen. A video camera placed on the top of the screen served to monitor and record the infant’s eye movements. A second experimenter, who was blind to the experiment, sat behind the monitor and recorded the looking time of the infant throughout the experiment. A third experimenter stroked the infant throughout the visuo-tactile condition. The infants could wear their bodysuit while being stroked.

All infants underwent two bimodal conditions (audio-visual and visuo-tactile) in separate blocks, one following the other. The order of presentation of each modality condition was fixed, i.e., all infants started with the visuo-tactile condition followed by the audio-visual condition. This choice was motivated by pilot testing, which showed that infants presented with the audio-visual condition first, would show a faster decay in attention in the visuo-tactile condition. To avoid obtaining confounding results in the visuo-tactile condition, we always presented the visuo-tactile condition first. A small break was allowed between the two bimodal conditions.

Within each bimodal condition, the infant underwent a habituation and a test phase. During the habituation phase, half of the infants were habituated to the congruent pairing (i.e., the two stimuli moving in the same direction), while the other half was habituated to the incongruent pairing (i.e., the two stimuli moving in opposite direction). Note that the direction of motion was counterbalanced across infants, so that, in the congruent pairings, half of the infants were habituated to a visuo-tactile or audio-visual stimulus moving upward, while the other half was habituated to a visuo-tactile or audio-visual stimulus moving downward. In the incongruent pairing, half of the infants saw a visual stimulus moving upward, accompanied with a tactile or auditory stimulus moving downward, and the other half saw a visual stimulus moving downward, accompanied with a tactile or auditory stimulus moving upward. Each habituation phase consisted of a maximum of 14 congruent or incongruent pairs of either audio-visual or visuo-tactile stimuli. An infant-controlled habituation paradigm was used. Each habituation trial was defined by the infant looking toward the stimulus for a minimum of 500 ms and was considered as terminated when the infant looked away for over 2 s. The habituation phase ended when the infant met an infant controlled criterion (i.e., a 50% decline in looking time on three consecutive trials, relative to the average looking time on the first three trials) or view all of the 14 congruent (or incongruent) bimodal trials.

Following each habituation phase, the infants were presented with a test phase that consisted of two pairs of test trials, each pair constituted by a congruent and incongruent trial (four test trials totally). As for the habituation trials, the minimum looking time required to be included in the final sample was 500 ms. There was no maximum duration of the trial, and looking times were recorded until the infant had a lookaway of 2 s. The test trials were presented sequentially, and the order of presentation of the trials was counterbalanced across infants, so that half of the infants habituated to the congruent movement was presented with a congruent trial first in the test phase (familiar trial) and the other half of the infants was presented with an incongruent trial (novel trial). For example, infants habituated to an upward congruent audio-visual stimulus could be presented with an upward congruent audio-visual stimulus first (familiar trial), followed by an upward auditory and downward visual stimuli second (novel trial). Direction of motion was counterbalanced too, so that *N* = 6 infants were habituated to an upward congruent visuo-tactile and audio-visual stimulus, *N* = 6 were habituated to a downward congruent visuo-tactile and audio-visual stimulus, *N* = 6 infants were habituated to an upward visual and downward tactile or auditory stimulus, and *N* = 6 infants were habituated to a downward visual and upward tactile or auditory stimulus.

Each test trial ended when the infant looked away for over 2 s.

All testing sessions were videotaped and subsequently frame-by-frame coded using a video capture/processing utility (VirtualDub) by one experimenter. In both the visuo-tactile and audio-visual condition, the coded onset of the looking times corresponded to the onset of the two stimuli (i.e., when they were temporally matched).

Twenty-five percent of the data were double-coded by a second coder who was blind to the conditions. In both conditions, a high level of agreement was confirmed between the two judges in their estimates on 25% of the trials (mean Pearson *r* = 0.90, *p* < 0.01, in both visuo-tactile and audio-visual condition).

### Results

The average looking times of all infants were log-transformed before being entered in a repeated-measures ANOVA (see Csibra et al., 2016).

**FIGURE 1:**
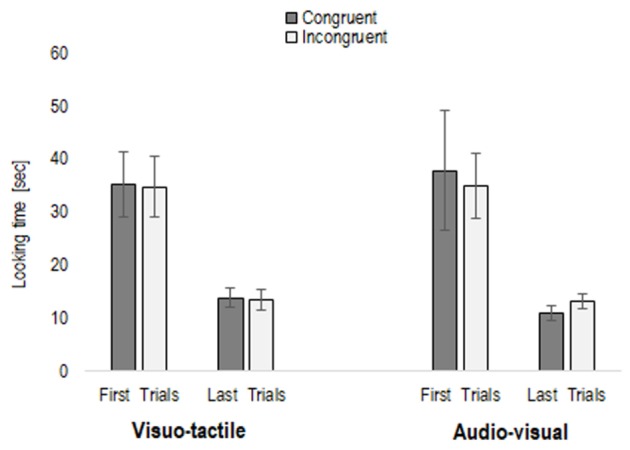
First three and last three trials in the habituation phase (congruent or incongruent stimuli), separately for the two conditions (visuo-tactile and audio-visual).

Once log-transformed, to observe whether infants habituated to the stimuli, we entered the average looking time of the first three and last three trials of the habituation phase into a repeated-measures ANOVA, with Trial (first three trials vs. last three trials) and Condition (visuo-tactile and audio-visual) as within-subjects factors, and Habituation type (congruent vs. incongruent habituation) as between-participants factor. This analysis revealed only a main effect of Trial, *F*(1,22) = 161.47, *p* < 0.001, η^2^ = 0.88, caused by average looking time on the first three habituation trials being significantly longer in comparison to the last three (mean first trials = 35.69 s, *SE* = 3.70; mean last trials = 12.84 s, *SE* = 0.83), irrespective of whether infants were habituated to a congruent or incongruent condition (*p* = 0.83), and whether the condition was visuo-tactile or audio-visual (*p* = 0.59) (see **Figure 1**). To further explore whether infants may present a different sensitivity to congruency, we entered the total looking time of the habituation phase in a repeated-measures ANOVA, with Condition as within-subject factor, and Habituation type as between-subject factor. However, no main effect nor interaction emerged (all *p* > 0.11), as infants watched the congruent habituation pairing (mean = 64.92 s, *SE* = 7.60) as much as the incongruent habituation pairing (mean = 61.12 s, *SE* = 7.60).

**FIGURE 2:**
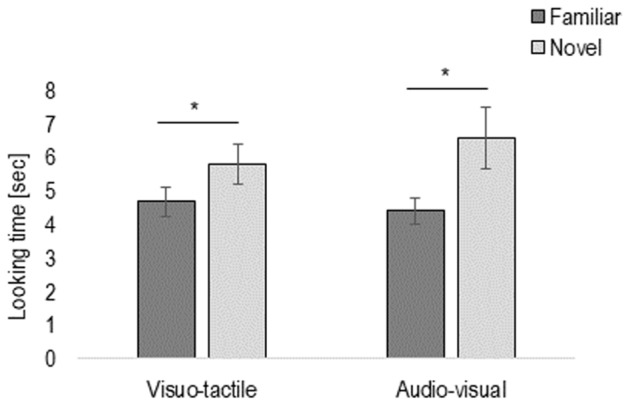
Results of Experiment 1: Total looking time of the test phase, presented here separately in the visuo-tactile and audio-visual condition. Error bars indicate standard error of the mean. Asterisks indicate a statistically significant difference between the novel and the familiar trial in both the visuo-tactile and audio-visual condition.

To investigate whether infants were able to discriminate the congruent vs. incongruent motion direction, we entered the looking time of the test trials into a repeated-measures ANOVA with Novelty (familiar vs. novel trial), Pair (first vs. second pair of familiar and novel trials), and Condition (visuo-tactile vs. audio-visual) as within-participants factor, and Habituation type (congruent vs. incongruent) as between-participants factor. The analysis revealed a main effect of Novelty, *F*(1,22) = 5.84, *p* = 0.02, η^2^ = 0.21, due to infants looking longer to the novel (mean = 6.18 s, *SE* = 0.64) than familiar stimulus (mean = 4.37 s, *SE* = 1.86), in both visuo-tactile and audio-visual conditions (*p* = 0.18), and irrespective of type of habituation (*p* = 0.11) (see **Figure 2**).

There was also a main effect of Pair, *F*(1,22) = 14.80, *p* = 0.001, η^2^ = 0.40, because of infants looking overall longer to the first (mean = 6.06 s, *SE* = 0.51) than the second pair (mean = 4.65 s, *SE* = 0.39) of familiar and novel test trials.

Overall, these results revealed that infants were able to discriminate between congruently and incongruently moving stimuli pairs. To further explore infant’s sensitivity to the congruency, we tested a new group of 3–4 month-old infants on a preferential looking paradigm in which congruent and incongruent stimuli were presented sequentially for a fixed time (maximum 60 s). The difference between this approach and the typical habituation paradigm is that the latter is commonly taken as evidence that infants are able to discriminate a novel stimulus on the basis of a pre-exposure (habituation) to a familiar/repeated stimulus. In Experiment 2 we measured whether infants spontaneously prefer a congruent over an incongruent stimulus by presenting only two trials in a within-subject design, in which infants had a fixed time to look to the stimuli presented.

## Experiment 2

### Method

#### Participants

Infants were recruited via a written invitation, which was sent to parents based on birth records provided by neighboring cities. We tested 16 infants (8 females; mean age = 109 days, range: 92–131 days) with no visual, auditory or tactile abnormalities, as reported by the parents on the questionnaire administered at the end of the testing. Additional four infants were tested but failed to conclude the testing because they were fussy (*N* = 2), or technical difficulties with the recording equipment (*N* = 2). No infant was at risk for developmental delay or disability (e.g., pre-term, low birth weight). Both parents signed an informed consent prior to the beginning of the experiment. The study was approved by the Ethical Committee of the University of Milan-Bicocca, and carried out in accordance with the provisions of the World Medical Association Declaration of Helsinki.

#### Materials

The stimuli were those used in Experiment 1.

#### Procedure

The infants sat on the lap of their mother who was blind to the experiment in a research room within the University of Milan-Bicocca. The recording parameters were the same as the ones used in Experiment 1.

All infants were presented with four trials: one congruent and one incongruent visuo-tactile trial, and one congruent and one incongruent audio-visual trial. The order of presentation of the trials (congruent or incongruent first) was counterbalanced across infants, so that half of the infants saw the congruent trial first, and the other half the incongruent trial. As in Experiment 1, all infants saw the visuo-tactile condition first, followed by the audio-visual condition. A small break was allowed between the presentation of the two conditions. In the visuo-tactile condition, the synchronous onset of the visual and tactile stimuli was determined by offline coding. Each trial had a maximum duration of 60 s, and it was presented until its end or until the infant looked away for over 5 s.

Again, as in Experiment 1, all testing sessions were videotaped and subsequently frame-by-frame coded using a video capture/processing utility (VirtualDub) by one experimenter. Twenty-five percent of the data were double-coded by a second coder who was blind to the conditions (i.e., whether the infants saw a novel or familiar trial first). A high level of agreement was confirmed between the two judges in their estimates on 25% of the trials (mean Pearson *r* = 0.90, *p* < 0.01, in both visuo-tactile and audio-visual condition).

### Results

As in Experiment 1, the average looking times of all infants were log-transformed before being statistically analyzed.

**FIGURE 3:**
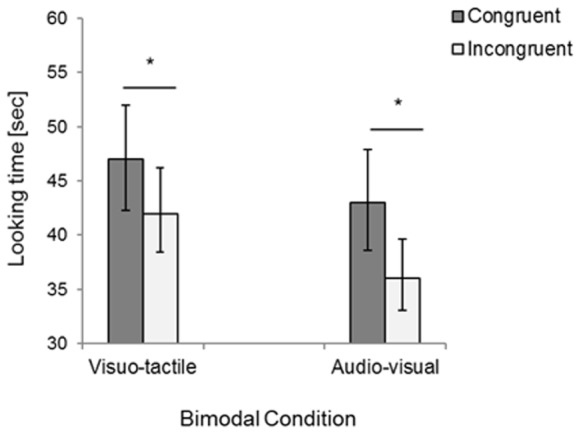
Results of Experiment 2: Total looking time for congruent and incongruent bimodal stimuli presented in the visuo-tactile and audio-visual condition. Error bars indicate standard error of the mean. Asterisks indicate a statistically significant difference between the congruent and the incongruent trial in both the visuo-tactile and audio-visual condition.

Total looking times were analyzed with a repeated-measures ANOVA, with Congruency (congruent vs. incongruent trials) and Condition (visuo-tactile vs. audio-visual) as within-participants factors. The analysis only revealed a main effect of Congruency, *F*(1,15) = 12.60, *p* = 0.003, η^2^ = 0.46, due to infants looking longer to the congruent compared to the incongruent trial in both sensory conditions (visuo-tactile congruent: *M* = 46.84, *SD* = 10.33, visuo-tactile incongruent: *M* = 41.82, *SD* = 11.73; audio-visual congruent: *M* = 42.55, *SD* = 12.56, audio-visual incongruent: *M* = 36.49, *SD* = 14.86, see **Figure 3**). Note that four infants watched the whole 60 s moving bar (*N* = 1 in the visuo-tactile congruent condition and *N* = 3 in the congruent audio-visual condition), while the looking times of the other infants ranged between 5 and 57 s before looking away for 5 s.

### Discussion

The present study showed that infants as young as 3–4 months of age are able to discriminate between direction of motion across different multisensory pairings (Experiment 1), and prefer congruent over incongruent multisensory moving stimuli (Experiment 2). The fact that infants are able to extract direction of motion from both sensory pairings suggests that motion itself may possess redundant characteristics that favor infant’s attention and thus the integration among senses. This would add evidence to the notion that amodal information promotes infants’ attention and perceptual learning during early development (Bahrick and Lickliter, 2000; Bahrick et al., 2004), and that motion itself is perceived as redundant across different sensory modalities. Because the onset of the stimuli was synchronous, it could be claimed that temporal synchrony – not motion – was the amodal component that favored the matching of the bimodal pairings. It could be that both information contributed to multisensory motion perception. However, temporal synchrony alone does not explain why infants preferred congruent over incongruent multisensory motion, as both the congruent and incongruent stimuli were synchronously presented.

The result of Experiment 2 revealed the most important and novel finding, that is, infants looked longer at a visual stimulus that is accompanied by a stimulus that moves in the same direction regardless of whether it moves in space or in tonal space. In fact, while perception of congruent motion direction is likely based on the shared physical properties of vision and touch (thus favoring the preference for a coherent motion), motion is not a physical property of sound.

The fact that infants preferred a congruent bimodal motion, irrespective of whether the motion was real or apparent also suggests that integration of illusionary and non-illusionary motion stimuli occurs very early in development. Indeed, the two conditions (audio-visual and visuo-tactile) differ in two respect, namely in integration of illusionary (audio-visual) and illusionary and real motion (visuo-tactile); this points, first, to the robustness of the effect observed, and generalizes across type of motion perceived (i.e., illusionary and non-illusionary). Second, it suggests that, particularly in the audio-visual condition, pitch may possess a spatial nature. In other words, the fact that infants preferred a low-to-high change in pitch with an upward moving visual stimulus, and a high-to-low change in pitch with a downward visual stimulus suggests that, in line with adult studies (Rusconi et al., 2006; Parise et al., 2014; Pitteri et al., 2017), pitch may be spatially represented, and that the association “high in pitch, high in space,” as well as “low in pitch low in space,” is learned very early in development.

With respect to previous literature, it should be noted that our findings appear only partially consistent with Bremner et al. (2011). Indeed, Bremner et al. (2011) found no spontaneous preference for audio-visual moving pairs up to 8 months of age. However, our stimuli were presented centrally on a vertical dimension, which could have facilitated the processing of such stimuli. This possibility is also corroborated by previous studies (see Walker et al., 2010; Dolscheid et al., 2014) that presented moving stimuli on a vertical dimension and found a spontaneous preference for audio-visual stimuli in 3 month-olds. It should also be noted that Bremner et al. (2011) found that, following habituation, even 2 month-olds detected the co-location of audio-visual stimuli, suggesting that, given enough time to process the stimuli, infants can learn to discriminate congruent from incongruent stimuli. Also, it could be argued that our 3 month-old infants both discriminated and preferred a congruent moving audio-visual and visuo-tactile stimulus because the stimuli we presented were particularly suited for the age. Indeed, the Barberpole illusion gives the impression of continuously moving in a sole direction, which – contrary to Bremner et al. (2011) – may have favored the processing of such stimuli.

A question that arises when looking at the two experiments is: why do infants habituated to congruent pairs did not show overall longer fixations than infants habituated to the incongruent condition? In other words, the spontaneous preference observed in Experiment 2 predicts longer fixations during the congruent habituation trials. However, this was not the case. Our suggestion is that the continuous repetition of the same trial during habituation may have flattened a potential preference. Indeed, In Experiment 1 we observed a decline in fixation that is determined by the infant (“infant controlled”) rather than by the experimenter, while in Experiment 2 spontaneous preference was assessed using a fixed trial duration.

Finally, some limitations of our study and suggestions for future ones should be underlined too. In fact, because in our study the stimulation was always bimodal, we cannot exclude that infants relied on one modality more strongly than the other to detect differences between the congruent and incongruent condition. Classical studies investigating the amodal property of stimuli have used different approaches than ours, for example the crossmodal transfer paradigm (e.g., Rose et al., 1981; Streri and Pêcheux, 1986; Sann and Streri, 2007), in which stimulation is first presented in one sensory modality, followed by presentation in another modality. This approach not only allows establishing whether a specific feature is transferred from one modality to another, but whether this transfer is bidirectional.

## Conclusion

In this study we showed that 3–4 month-old infants can discriminate between different directions of motion in visuo-tactile and audio-visual compounds. Such discrimination is accompanied by a general preference to coherent vs. incoherent motion across senses. It could be speculated that specific neural structures predispose the infant to bind specific properties of the stimuli. For instance, studies conducted in primates have found directionally sensitive neurons particularly in the posterior parietal cortex, which is a multisensory convergence site, receiving projections from the visual and somatosensory motion areas, as well as being responsive to spatial properties of auditory stimuli (Schlack et al., 2005). In other words, it could be that specific, directionally sensitive neurons in multisensory areas that are frequently activated because of the (multisensory) experiences of the infant promote an early development of motion sensitivity across senses, and future studies might further investigate this line of inquiry.

## Author Contributions

EN, MG, and CT designed the experiment. EN, VB, and EC acquired data. EN analyzed the data. All authors contributed to the interpretation of the data, and approved the final version of the manuscript.

## Conflict of Interest Statement

The authors declare that the research was conducted in the absence of any commercial or financial relationships that could be construed as a potential conflict of interest.
